# Managing the water-energy-food nexus in the adige river basin: impacts of climate and land use change on ecosystem services bundles

**DOI:** 10.1007/s10584-025-04013-3

**Published:** 2025-09-11

**Authors:** Beatrice Sambo, Anna Sperotto, Celina Aznarez, Stefano Terzi, Massimiliano Pittore, Andrea Critto, Silvia Torresan

**Affiliations:** 1 Department of Environmental Sciences, Informatics and Statistics, University Ca’ Foscari Venice, I-30170 Venice, Italy; 2 Fondazione Centro Euro-Mediterraneo sui Cambiamenti Climatici, Lecce, I-73100 Italy; 3 Eurac Research, Center for Climate Change and Transformation, Bolzano, 39100 Italy; 4 Basque Centre for Climate Change (BC3) Scientific Campus of Basque Country, Leio, Building 1, 1 st floor, Barrio Sarriena s/n, Bizkaia 48940 Spain; 5 Section for Ecoinformatics and Biodiversity, Department of Biology, Aarhus University, Aarhus, DK-8000 Denmark

## Abstract

The Water-Energy-Food (WEF) nexus framework highlights the interdependencies among water, energy, and food systems. Integrating Ecosystem Services (ESs) enhances this approach by incorporating ecological benefits for more holistic assessments. This study applies a spatially explicit ESs-based WEF nexus analysis in the Adige River Basin (Northern Italy), focusing on five ESs: water provisioning, crop yield, sediment retention, carbon storage, and landscape diversity, under two future scenarios (SSP1-RCP 2.6 and SSP5-RCP 8.5) from 2018 to 2050. Using Self-Organizing Maps, sub-basins were clustered into ESs bundles enabling the identification of tailored management strategies. Results reveal spatial heterogeneity and shifts in ESs bundles, with synergies often found in upstream, forested areas. Under high-emission scenarios, regulating services decline and provisioning services face trade-offs, especially with intensified agriculture. Key strategies suggested include maintaining environmental flows, reducing synthetic fertilizers, promoting reforestation, crop diversification, and expanding protected areas. These are structured into physical, economic, and climatic pathways aligned with EU restoration goals. Findings demonstrate the value of an ESs-bundles approach for optimizing synergies and managing trade-offs across the WEF nexus.

**Supplementary Information:**

The online version contains supplementary material available at 10.1007/s10584-025-04013-3.

## Introduction

The impacts of climate and land use changes on natural resources are becoming increasingly evident, driven by rising global demand for water, energy, and food. This intensifies pressure on ecosystems and underscores the need for integrated resource management, as c.a60% of Ecosystem Services are already partially or fully degraded due to unsustainable land use practices (Vihervaara et al., 2010). Ecosystem Services (ESs), defined as the benefits that ecosystems provide to people, have evolved from a conceptual tool to an applied framework shaping sustainability science, policy and spatial planning (Díaz et al. 2018). ESs assessments often distinguish between supply (i.e. the ecosystem’s capacity to provide services), demand (i.e. the societal need for those services) and flow (i.e. the actual benefits received by people), covering the multiple perspectives on human–nature interactions (Villamagna et al. 2013; Schröter et al. 2014).

In parallel, the Water-Energy-Food (WEF) nexus has become a critical framework for understanding and managing interdependent resource systems. Originating from international policy dialogues (i.e. 2011 Bonn Conference), the WEF nexus emphasizes the interlinkages between water, energy and food security, together with the need to consider them in governance and planning. Integrating ESs into the WEF nexus strengthens its ecological grounding by incorporating key ecosystem functions such as nutrient cycling, carbon sequestration, and water filtration, that sustain the natural capital underpinning resource flows (Lucca et al. 2025; Sambo et al. 2024a, b; Hoff et al. 2019). This integration enhances the capacity to address trade-offs, maximize synergies, and mitigate ecosystem degradation (Sambo et al. 2024a, b), while supporting broader goals like climate resilience, biodiversity conservation, and equitable access to resources, which are key aims of the UN Sustainable Development Goals (United Nations 2019).

Population growth, extreme climate events, and land-use disturbances are intensifying pressure on natural resources, requiring an integrated understanding of interactions within the WEF-ESs framework (Lucca et al. 2025). Effective nexus management goes beyond analyzing ESs relationships, like water availability, and resource use, by balancing these demands with environmental integrity and human well-being (Bidoglio et al. 2018; Shah 2023). Meeting projected needs, like a 50% increase in agricultural production and 30% more water withdrawals by 2050 (Wang et al. 2024), is especially challenging given that 2.4 billion people already face water stress and 40% of croplands experience scarcity (FAO 2021); Liu et al. 2022; Vihervaara et al. 2010). While increased demands present economic opportunities, particularly in developing regions, they also heighten pressure on strained water and energy systems, emphasizing the need for sustainable management practices (Santos et al. 2023).

As water moves through landscapes, it shapes eco-hydrological dynamics, influencing ESs provision and broader ecosystem processes. Climate change further alters these dynamics, necessitating adaptive management and long-term planning to address local-scale impacts with precise, context-specific climate information (Aznarez et al. 2021). Mountain ecosystems, such as those in the Adige Basin in Northern Italy, exemplify these dynamics. Characterized by complex terrain, steep altitudinal gradients, and high biodiversity, the Adige is one of the major alpine catchments in Italy and plays a critical role in regional water, energy, and food systems. These highland regions act as natural water towers (Pereira et al. 2022), regulating water availability downstream while supporting key ESs such as carbon sequestration, soil retention, and recreational value (Schirpke and Ebner 2022; Gratzer and Keeton 2017). Like many other mountainous regions in Europe, the Adige River basin is undergoing substantial land-use transformation driven by socio-economic, climatic, and environmental factors (Gaglio et al. 2020). These changes arise both from natural processes, such as ecological succession after land abandonment or climate-driven shifts, and from human interventions including urban expansion and land reclamation. Such transformations increasingly threaten ESs in mountain regions (Bilbao-Barrenetxea et al. 2024; et al. 2023). While some ESs, like food production and forest-related functions (e.g., carbon sequestration, pollination, soil formation) may experience localized benefits, many regulating and cultural services are adversely affected (Egarter Vigl et al. 2021; Schirpke et al. 2012). A spatial gradient in ES supply and demand is evident across Alpine landscapes like the Adige Basin. Sparsely populated upstream areas with extensive forest cover predominantly supply regulating and cultural services, while downstream valleys, with denser populations and intensive land use, are characterized by higher demand for provisioning services such as irrigation, food, and hydropower (Schirpke et al. 2012, 2019). These patterns create interdependencies across the landscape and highlight potential mismatches that may intensify under future environmental change. In regions like the Alps, steep gradients and land tenure mosaics contribute to high spatial heterogeneity in ESs provision (Schirpke et al. 2012), making it a representative case for understanding how WEF-related ESs interact under climate and land use change pressures. Disentangling these interactions is essential for developing strategies that mitigate negative impacts. Changes in agricultural policies, market prices, or land use often drive resource users to adjust their extraction from the environment and usage of ESs, often adopting unsustainable practices in response to external pressures (Pacheco et al. 2025; Carrer et al. 2020). In mountain basins like the Adige, upstream management decisions can strongly influence downstream ecosystem functions and socio-economic resilience (Carrer et al. 2020; Nepal et al. 2014). Addressing these cross-scale interactions requires integrated analyses of climate, land use, and economic factors to formulate effective WEF nexus strategies (Chen et al. 2022; Yin et al. 2023).

In this context, the concept of ESs bundles provides a systems-level perspective that complements traditional ESs assessments. ESs bundles are recurring sets of services that co-occur in space and time due to shared ecological, climatic, or socioeconomic drivers (Raudsepp-Hearne et al. 2010; Vannier et al. 2019). Unlike assessments centered on individual ESs or mismatches in supply and demand, the bundle perspective emphasizes interactions, highlighting synergies (i.e. where ESs increase or decline together) and trade-offs (i.e. where gains in one service may reduce others) (Vallet et al. 2018; Turner et al. 2014). Identifying these bundles supports recognizing multifunctional landscapes and help balancing competing land uses across the WEF sectors (Vannier et al. 2019). While traditional assessments often use administrative boundaries for data convenience, these rarely align with ecological processes. Sub-basins offer a more ecologically meaningful unit, bridging biophysical features with human management (Pacheco et al. 2025; Zhang and Wang 2024; Aznarez et al. 2021). Given that ESs are highly responsive to climate and land-use changes, an integrated systems perspective is essential to account for trade-offs and synergies in their management (Sambo et al. 2024a, b). Examining ESs associations in areas with similar geographic characteristics further improves understanding of their consistency and interrelationships (Reader et al. 2024; Mouchet et al. 2017). Static ESs bundles analyses have informed land use management (Dou et al. 2020) but incorporating both current and historical ESs dynamics can better capture evolving interactions and support long-term sustainable management (Gou et al. 2022; Yang et al. 2021a, b; Han et al. 2021). Tools like Self-Organizing Maps (SOM) efficiently handle large datasets and spatial complexity, making them well-suited for exploring ESs synergies and trade-offs across scales (Mouchet et al. 2017; Cord et al. 2017; Dou et al. 2020; Xia et al. 2023).

This study analyzes temporal and spatial changes in ESs interactions within the Adige Basin under future climate and land use change scenarios. Using the WEF nexus framework and insights from the NEXOGENESIS project (Sambo et al. 2024a, b), it offers actionable recommendations for enhancing ES bundles. The objectives are to: (i) assess sub-basin scale heterogeneity of WEF nexus-related ES bundles, (ii) model future climate and land use impacts, and (iii) support spatial planning and WEF management at the sub-basin scale.

## Case study

**Fig. 1:**
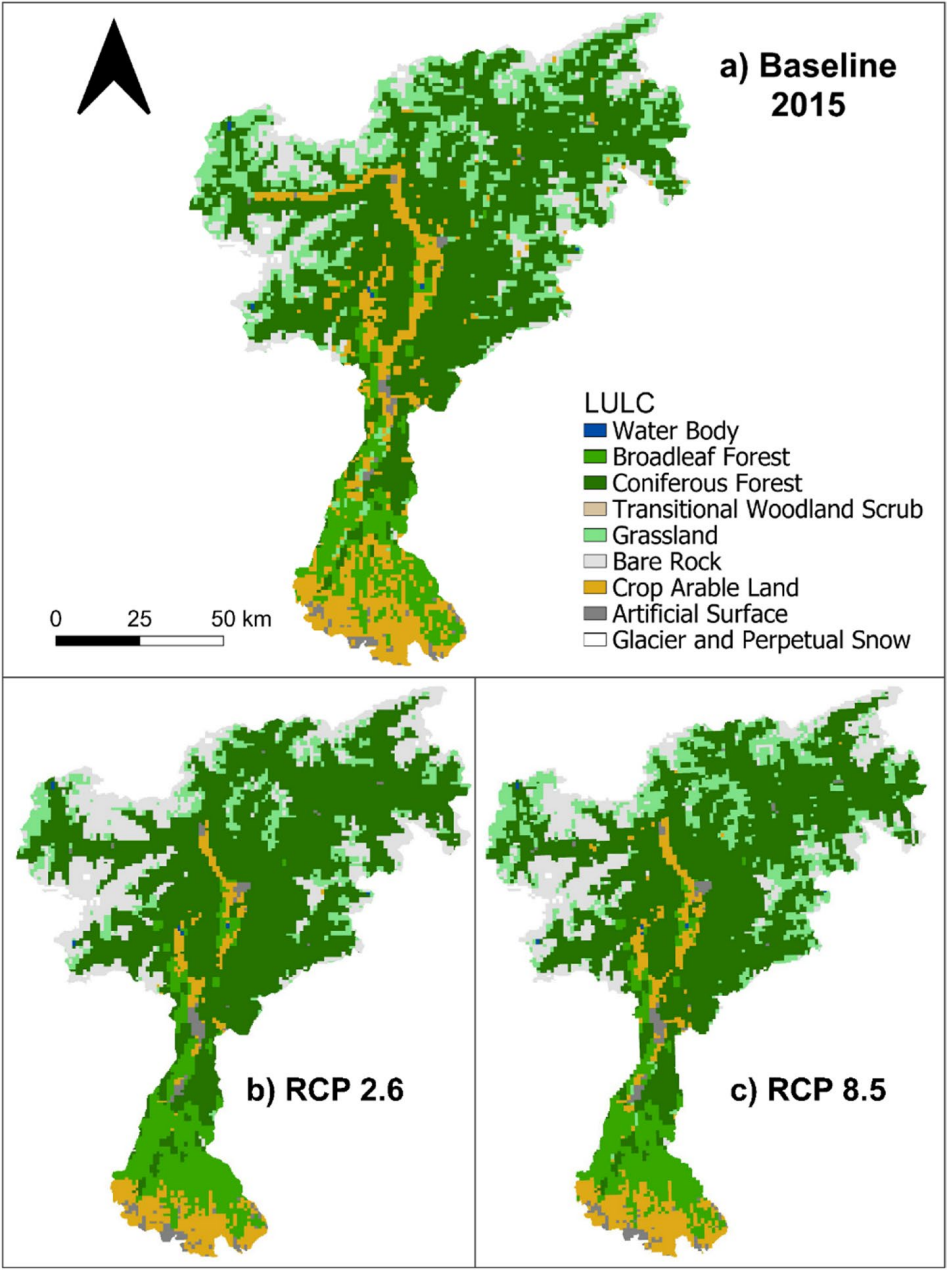
Case study area with baseline land cover (**a**), and two future scenarios for 2050: SSP1-RCP 2.6 (**b**) and SSP5-RCP 8.5 (**c**).Compared to the baseline, SSP1-RCP 2.6 shows reductions in arable land, grasslands and urban expansion, and increases in forested area and perpetual snow while SSP5-RCP 8.5 depicts more pronounced land-use changes, including further loss of snow cover and increased arable land and urban sprawl, particularly in valley areas. These shifts illustrate the contrasting socio-economic and climatic trajectories shaping ecosystem services provision

The Adige River basin, Italy’s third-largest catchment, spans approximately 12.100 square kilometers, covering parts of the provinces of Bolzano, Trento, Verona, Vicenza, Belluno, Padua, Rovigo, and Venice. This basin is characterized by a diverse landscape, ranging from the high-altitude, mountainous regions of Trentino-Alto Adige to the flat, low-lying plains of the Veneto region (Fig. 1).

The upper part of the basin is a typical high-altitude mountain landscape, dominated by glaciers, bare rock and rugged terrain. As elevation decreases, the landscape transitions into extensive coniferous and broadleaf forests, interspersed with grasslands, which further shape the ecological dynamics of the region. This diverse land cover plays a crucial role in regulating river flow, supporting biodiversity, and providing essential ecosystem services such as water purification, flood regulation, and habitat provision for diverse species. Additionally, the forests and grasslands contribute to carbon sequestration, soil retention, and slope stabilization, helping to mitigate erosion and landslides while sustaining the overall environmental balance of the basin.

As the river descends, the terrain flattens into primarily agricultural areas before reaching the delta. In these areas, agriculture relies heavily on river water for irrigation, particularly in the upstream mountain valleys. These fertile areas are well known for intensive apple orchards, contributing over 15% of European apple production (FAO 2021) as well as for fruit berries, olive groves and wine production. The need to manage water resources sustainably is critical to maintaining food security while avoiding conflicts between agricultural and other water uses. The Adige River in fact is crucial for various water uses, including drinking water, irrigation, hydropower, industrial processes and tourism (Braioni et al. 2002; Egarter Vigl et al. 2016; Mozzi et al. 2020). Hydropower is a major component of the energy matrix in the Adige River basin, particularly in the upper part, where 61 hydropower stations generate more energy than is locally consumed (Terzi et al. 2021).

Figure 1 show expected land use for the case study area according with the land use projection of Chen et al. (2022) considering baseline condition at 2015 and two future scenarios for 2050 (i.e. SSP1-RCP 2.6 and SSP5-RCP 8.5). Future projections for the Adige River basin indicate significant land-use transformations under both future scenarios. Grassland cover in the northern part of the basin is expected to decline, primarily due to ongoing land abandonment and changes in climatic conditions. Similarly, agricultural land extension at higher elevations is anticipated to decrease. This trend is especially prominent at higher elevations and in less accessible locations, where traditional and small-scale farming will become increasingly unprofitable compared to agricultural activities in lower-altitude areas (Bonari et al. 2025; Gobbi 2021; Faccioni et al. 2019). For the same reason, a slight increase in agricultural areas may occur in the central, more profitable, parts of the basin (Mascetti et al. 2023). Forested areas are projected to expand throughout the basin, largely because of natural succession processes in abandoned land. At the same time, bare land cover in the northern regions is expected to increase. As low-intensity farming and grazing practices diminish, previously vegetated land is left exposed and becomes more vulnerable to soil erosion. In combination with rising temperatures and glacial retreat, as well as shifts in vegetation dynamics at high altitudes, it could contribute to the expansion of barren rock surfaces. Urban expansion is also expected, with artificial surfaces slightly increasing, particularly around major urban centers such as Trento and Bolzano, driven by infrastructure development and population dynamics.

## Methods

**Fig. 2:**
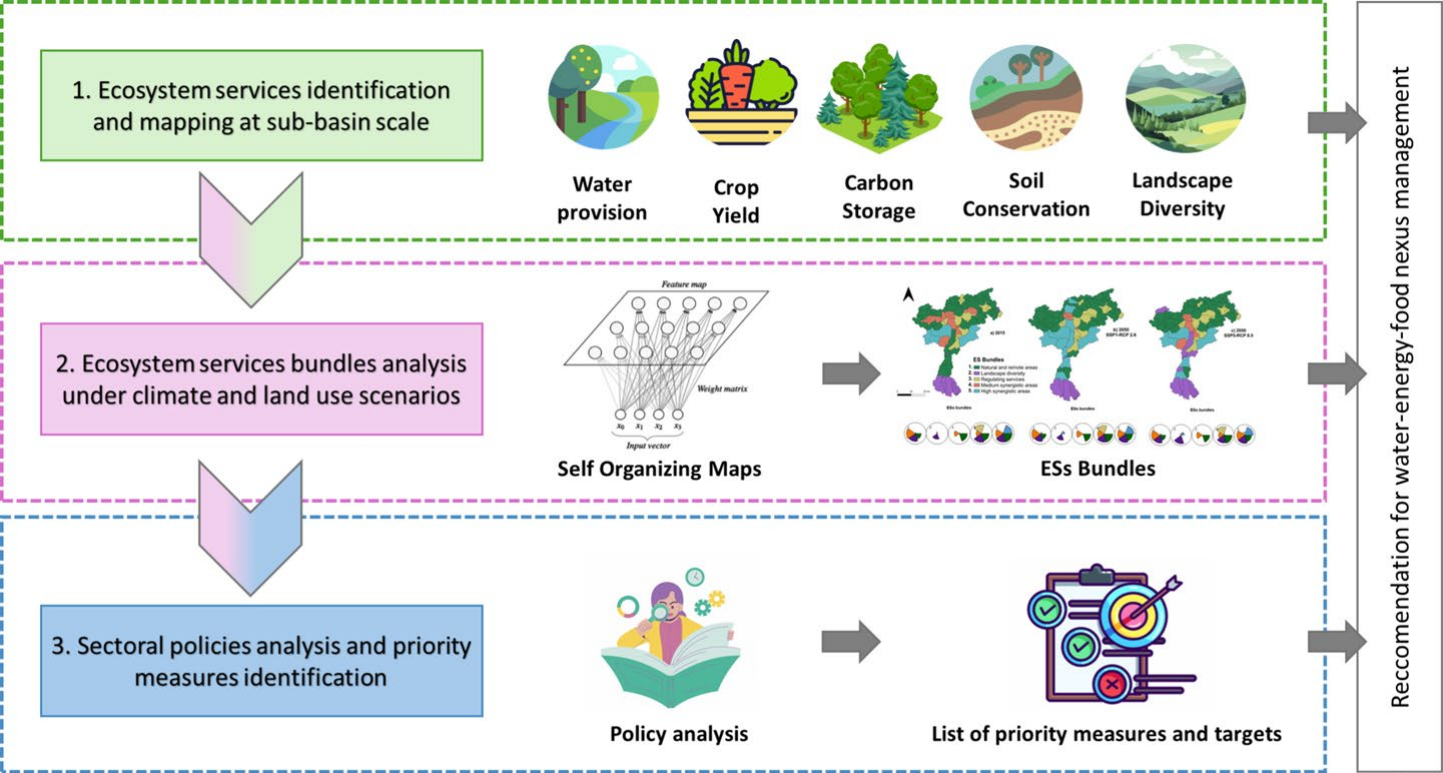
General methodological framework adopted in the Adige River basin

The methodological approach proposed in this work seeks to adopt an Ecosystem Services-based perspective to the assessment of the impacts of climate and land use changes on the WEF nexus in the Adige River Basin (Italy). As describe in Fig. 2, it integrates (i) the identification and mapping of ESs sustaining the WEF nexus in the case study; (ii) a spatially-explicit assessments of ESs bundles dynamics under alternative future scenarios; (iii) the analysis and recommendation of priority interventions for improving overall nexus sustainability.

### Data collection

Multisource datasets were utilized for the spatial assessment of ESs (Table 1) under current and future scenarios. The highest possible resolution was chosen for each data type, with a preference for local sources wherever available.

**Table 1:** Data implemented in the analysis and related data source

Data type	Units	ESs	Data source	Spatial resolution
Land use	M	CY, RV, CS, SI	(Chen et al. 2022)	1 km
Runoff	M^3^/s	WP	CMIP6- MPI-model	-
Precipitation	Mm	WP	CMIP6- MPI-model	-
Temperature	°C	WP	CMIP6- MPI-model	-
Soil C storage	t/ha	CS	SoilGrids-ISRIC	250 m
Type of Eco-floristic region	--	CS	FAO	-
Type of Continental region	--	CS	Continental region classification (Oak Ridge National Laboratory)	-
Future rainfall erosivity (R-factor)	MJ-mm ha-1 h-1 year-1	RV	ESDAC JRC	500 m
Soil Erodibility (K- Factor)	t ha h ha-1 MJ-1 mm-1	RV	ESDAC JRC	500 m
LS-factor (Slope Length and Steepness factor)		RV	ESDAC JRC	100 m
Cover management (C-factor)	(0–1)	RV	(Panagos et al. 2015a, b)	National
Support practice (P-factor)	(0–1)	RV	(Panagos et al. 2015a, b)	National
Crop yield	(t/ha)	CY	ISTAT	Provincial

This study considers three land cover scenarios: a baseline period (2015) and two future projections for 2050 based on Shared Socioeconomic Pathways (SSP) (SSP1-RCP 2.6 and SSP5-RCP 8.5) (Fig. 1). Five ESs indicators were selected based on data availability and relevance to the scenarios. The chosen SSPs are: SSP1: Sustainability (Taking the Green Road), and SSP5: Fossil-fueled Development (Taking the Highway), represent contrasting yet optimistic development trajectories. Both assume strong investments in education, healthcare, and the establishment of strong institutions. However, SSP1 envisions a gradual transition to sustainable practices, while SSP5 relies on fossil-fuel-based, energy-intensive economy (Rakhmatova et al. 2024). These scenarios were used in the IPCC Sixth Assessment Report to model five different scenarios (SSP1-SSP5)tof temperature outcomes by 2100, each associated with anticipated levels of radiative forcing by 2100 (ranging from 1.9 to 8.5 W/m²) (Rakhmatova et al. 2024). SSP1-2.6 scenario reflects low greenhouse gas (GHG) emissions, aiming for net zero CO2 emissions by 2075, whereas SSP5-8.5 reflects very high GHG emissions, with CO2 emissions expected to triple by 2075. These two scenarios were selected as the study’s ‘best case’ (SSP1-2.6) and ‘worst case’ (SSP5-8.5) scenarios. To simulate future land use scenarios, we utilized LULC data (1 km-resolution) for 2015 and projections for 2050 under SSP1-RCP 2.6 and SSP5-RCP 8.5, as developed by Chen et al. (2022). Climate projections data (i.e. temperature and precipitation) for the future were retrieved from the IPCC based on the Coupled Model Intercomparison Project (CMIP6), using the MPI model, which was deemed the most appropriate for this analysis (Tebaldi et al. 2021). Data characterizing the R factor have been retrieved from ESDAC JRC database.

### Ecosystem services mapping

Five ESs representing each component of the WEF nexus were selected for mapping based on data availability and the characteristics of the case study area. These include water provisioning (WP), crop yield (CY), sediment retention (RV), carbon storage (CS), and landscape diversity (SI). Each ESs was mapped at the sub-basin scale for both the baseline and future scenarios using specific sectoral models and tailored data sources as described below (Table 2). More information related to single ESs can be found in the Supplementary Material.

**Table 2:** Ecosystem services and indicators assessed in this study

WEFE Sector	Ecosystem Service	Indicators	Abbreviation	Description	Model/source
Water	Water Provisioning	Runoff (m3/s)	WP	Availability of water to produce energy and food, for the sustainment of different ecosystems.	TOPMELT 1.0 model (Zaramella et al. 2019)
Food	Crop yield	Orchards yield (t/ha) Vineyards yield (t/ha) Maize/Cereals yield (t/ha)	CY	The quantity of agricultural production obtained from crops; contributes to sufficient food security.	Corine land cover ISTAT
Food-Ecosystems-Water	Sediment retention	Soil retained by vegetation (t/ha)	RV	The amount of soil retained by vegetation prevents the erosion and the dispersion of soil into water bodies, affecting water and ecosystem quality.	RUSLE (Ruesch and Gibbs 2008)
Energy	Carbon storage	C organic mass (kg)	CS	The amount of carbon stored in the soil, sequestrating carbon dioxide (CO_2_) from the atmosphere; contributes to regulating and mitigating climate change.	Ruesch and Gibbs 2008
Ecosystems	Ecosystem type diversity	Shannon Index (0–1)	SI	The diversity of types of ecosystems, describing the variety of a landscape gives importance to ecosystems and natural areas.	Shannon Index R

#### Carbon Storage - CS

Carbon storage was calculated using the ARIES (Artificial Intelligence for Ecosystem Services) (https://aries.integratedmodelling.org)/Tier 1 carbon models (Martínez-López et al. 2019). ARIES is AI-powered platform for data and model integration used to developing customizable ES models. Tier 1 model incorporates global lookup tables for vegetation carbon storage (Gibbs and Ruesch 2008), and spatially explicit global soil carbon storage data from ISRIC- World Soil Information [https://www.isric.org/explore/soilgrids]. Total ecosystem carbon storage is computed as the sum of the carbon mass stored in aboveground and belowground vegetation, plus the amount of carbon stored in the first 200 cm of soil. This study used the organic carbon mass (kg) as the indicator. To ensure comparability with other ESs, pixel-level results were aggregated at the sub-basin scale using a Zonal Statistic analysis with the mean value for each sub-basin in the years under study.

#### Crop Yield - CY

Crop yield was calculated using land cover maps to extract cropland areas, following the methodological process of Xia (et al. 2023) processed in R with the “sf” and “terra” packages. The resulting raster data were converted to sub-basin-level estimates using QGIS’s Zonal Statistic “count” function, allowing for comparison with other ESs. Additionally, crop yield data from ISTAT (National Statistical Institute) at the provincial level were assigned to sub-basins within the provinces of Bolzano, Trento, and Verona) (ISTAT 2024).

#### Water Provisioning - WP

Water provisioning was modeled using the ICHYMOD hydrological model, which combines the TOPMELT snowpack model from with a conceptual rainfall–runoff hydrological model at the basin scale. This model converts snowmelt and excess precipitation into runoff at the basin outlet and includes a snow routine, soil moisture routine, and flow routine (Shrestha et al. 2023; Zaramella et al. 2019). This method was applied to the Adige River basin, providing detailed runoff predictions. Monthly runoff data were obtained, and average values for June, July, and August (JJA) were used for analysis for 2015 and the two future scenarios for 2050 (Shrestha et al. 2023; Zaramella et al. 2019). These months were selected due to their critical importance from a nexus perspective, as June-July-August typically coincide with peak temperatures, reduced precipitation, and the highest irrigation demands in the Adige River basin, reflecting both climatic stress and intensive agricultural activity.

#### Sediment retention - RV

Sediment retention was calculated using the k.Lab software from ARIES, which applies the Revised Universal Soil Loss Equation (RUSLE; Renard et al., 1997) to estimate soil loss and retention by vegetation (RV) in tons of sediment per hectare per year (Martínez-López et al. 2019). The potential value (supply) of the sediment regulation ES is assessed by calculating RUSLE in two steps: first, using the most accurate available land cover data, and then, replacing all land cover with bare soil. The difference between these results estimates the amount of soil erosion prevented by vegetation (Martínez-López et al. 2019). Results were initially generated at the pixel level and subsequently aggregated to the sub-basin scale to allow for comparison with other ESs. A Zonal Statistic analysis was performed in QGIS, with the mean value (t/ha) of soil retention used for each sub-basin.

#### Shannon diversity Index – SI

To represent the ecological dimension of the nexus, we calculated the Shannon diversity index (SI) on the distribution of ecosystem types within each spatial unit. This index captures the heterogeneity of ecosystems, which is considered to influence the landscape’s capacity to simultaneously provide multiple ESs, both directly and indirectly related to the nexus (Mouchet et al. 2017; Alsterberg et al. 2017; Stürck and Verburg 2017).

The index quantifies the diversity of ecosystems types, where lower values mean that ecosystems type pixels belong to the same class, and higher SI refers higher heterogeneity of ecosystems (Legarreta-Miranda et al. 2021; Tonetti et al. 2023). The SI was calculated starting from ecosystem type maps that were derived incorporating the classification from the IUCN Global Ecosystem Typology with data about temperature and land cover from detailed land cover maps using packages “sf”, “terra”, “ggplot2” and “viridis” in R software.

### Ecosystem services bundles for Spatial management and planning strategies

After single ESs have been mapped, an analysis of ESs bundles was performed. ESs bundles refer to groups of multiple ESs that consistently co-occur in space and time due to shared ecological, climatic, or socio-economic drivers.

#### Ecosystem services bundles

ESs bundles were developed using Self-Organizing Maps (SOM), an unsupervised learning spatial neural network method that clusters ESs based on their spatial co-occurrence and similarities (Willighagen et al. 2007). Single ESs values were normalized among them between 0 and 1, with minimum-maximum normalization, before applying SOM to facilitate the comparison of diverse data sets. The SOM analysis was conducted using the “Kohonen” package (Willighagen et al. 2007) in R software, which provides tools for training and visualizing self-organizing maps. This allowed for the identification and visualization of ES bundles under the baseline and future scenarios, providing evidence to inform spatial management and planning strategies. To determine the correct number of bundles, different tests have been conducted alternatively setting the maximum number of clusters; upon examining the characteristics of these bundles there were similarities that could represent redundancy. Manually testing bundle numbers, and observing the results for each, it was possible to understand how to maximize the differences between bundles while keeping the total number of bundles as low as possible. For an in-depth description of the different tests performed and alternative bundles configuration considered please see Annex I).

#### Management measures identification and selection

Local sectoral policies aimed at sustaining the future of ESs targets were identified and analyzed through an engagement approach with local stakeholders conducted in the frame of NEXOGENESIS project. Different engagement activities have been carried out to explore how local stakeholders perceived the theme of WEF nexus and ESs within the Adige River basin and based on their sector of expertise (Sambo et al. 2024a, b). Stakeholders have been asked to provide opinion and suggestion of different measures that could be implemented in the context of WEF nexus to sustain ESs in a short-term future period. Moreover, an analysis of sectoral local policies has been conducted, to identify future targets to be selected for ESs sustainment. The policies linked to the ESs in the Adige River basin were prioritized, focusing on strategies to enhance their provision; additionally, policies from various sectors that target ecosystems and land use components were reviewed to identify potential measures for future scenarios. These measures are intended to support the ES component across multiple sectors. The policy measures were then categorized into three main types: (i) regulatory, (ii) market-based, and (iii) incentive-driven approaches. For each measure, specific targets were derived, based on the goals reported in the respective policies and the perspectives shared by stakeholders.

For each ESs bundle, based on the targets identified, measures have been suggested. The aim was not to recommend transitioning from one bundle to another but rather to determine the appropriate measures and targets for preserving each bundle, allowing for the simultaneous management of interacting ESs. Results of this will be presented in the following section, where for each developed bundle the characteristics and measures’ suggestion are described.

## Results

### ESs mapping

ESs mapping showed varying results both at the ESs level and across the years, reflecting both climate and land use changes. Highest values of Carbon sequestration (**CS**) supply can be found in the northern and central parts of the basin, where extensive forests and grasslands facilitate carbon sequestration- Conversely, agricultural land in the southern part of basin exhibit lower CS values due to the intensive farming and monoculture practices, which reduce organic matter input and limit long-term. CS remained relatively consistent across the different scenarios. Only a small decrease, most pronounced for the RCP8.5 scenario, can be detected in the high-altitude sub-basins located in the north-west part of the case study interested by the higher loss of vegetated areas.

**Fig. 3:**
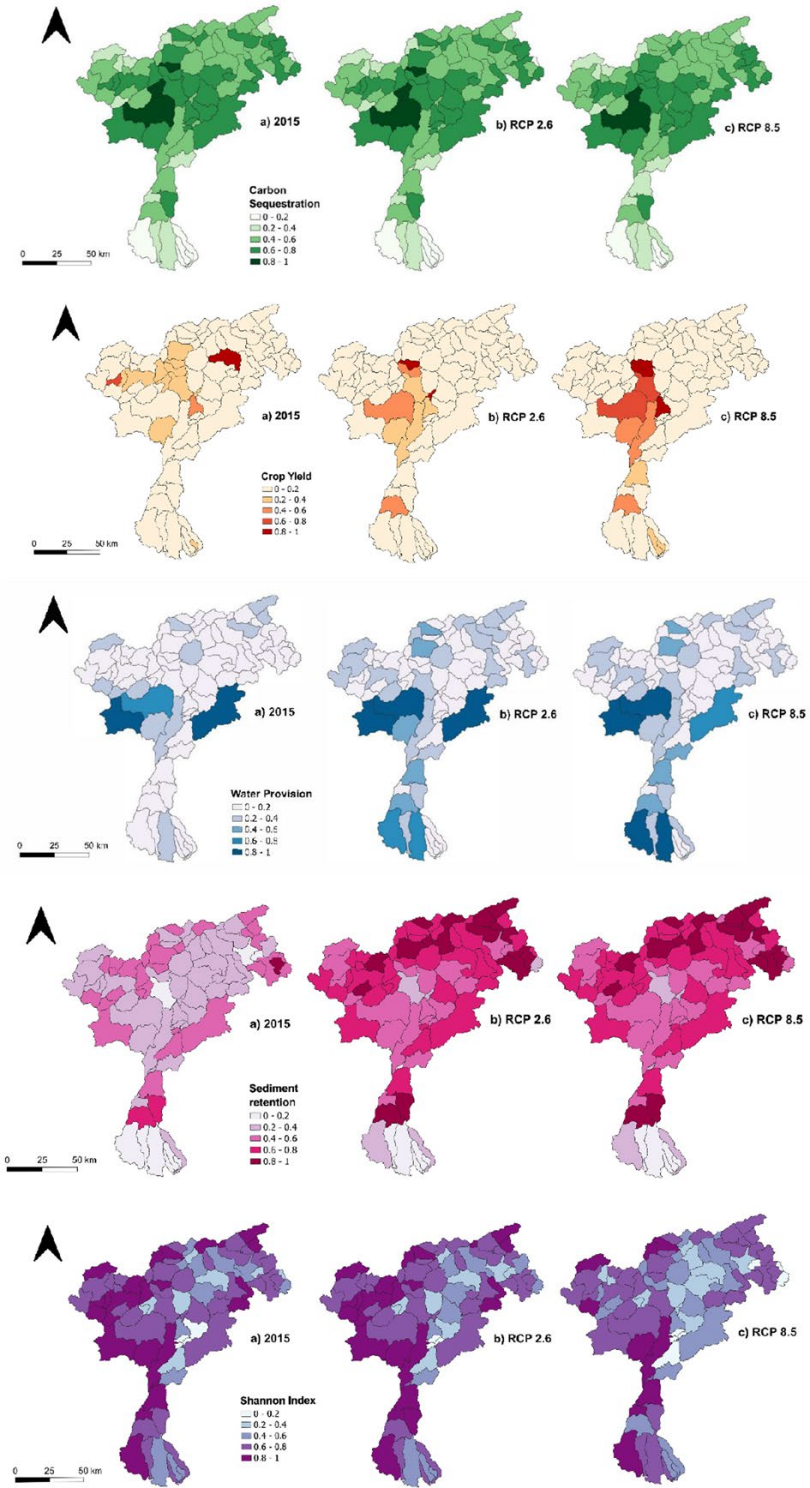
Ecosystem Services spatial distribution for baseline (2015) and two future scenarios: 2050 SSP1-RCP2.6 and 2050 SSP5-RCP8.5

High values of Crop yield (**CY**) in the baseline are located mainly in the northeastern part of the study area, characterized by orchids and berries plantation. However, CY is the service that varies the most across scenarios; although future scenarios project a reduction in the overall extension of agricultural areas (Fig. 1), CY exhibits temporal variability. This is primarily due to the expansion of orchards and vineyards, which are the main contributors to crop yield and increase their influence on total yield over time. Across the two scenarios, it is possible to observe that the change of distribution at the sub-basin level is heterogeneous, however, how CY in SSP5-RCP8.5 has higher values in the northeastern area, where higher cropland extension is detectable, and it is likely related to the increase of vineyards or orchards which contribute greater to increase CY. Moreover, from Fig. 1 it can be seen that croplands are identifiable also in the central part of the case study in higher extension in SSP5-RCP8.5 compared to SSP1-RCP2.6; this is reflected in ESs patterns results (Fig. 3) where CY has higher values in the SSP5-RCP8.5 scenario. According to Egarter Vigl (et al. 2016), vineyards and orchards are expanding replacing annual crops such as grains, having a higher yield, this influences the total CY detectable in this analysis.

Higher Water provisioning (**WP**) values are concentrated in the central and northeastern parts of the basin, characterized by higher elevations and by the presence of glaciers, covered by dense forest and grasslands primarily. In these areas, WP is strongly influenced by the snowmelt, which significantly contributes to water flow. On the other hand, in sub-basins mainly covered by bare rock or glacier retreat zones, WP is lower. In future scenarios, WP values increased in the southern areas due to changes in precipitation and temperature patterns. For each future scenario, changes are detectable, both in the north-central area of the case study and particularly in the southern area close to Verona. Between 2015 and the SSP5-RCP8.5 scenario, significant changes are taking place; notably, there is a substantial increase in WP in the southern region, likely driven by changing climatic conditions (i.e. increasing precipitation) or land use changes (i.e. agricultural expansion due to anthropogenic activities).

For the baseline the highest Soil retention by vegetation (**RV**) values are recorded in densely forested sub-basins. Low RV values occur in high-altitude barren zones and intensively farmed southern croplands, where limited vegetation leads to higher runoff and erosion. As shown in Fig. 3, future scenarios project increased RV, particularly in central and northwestern sub-basins, due to vegetation expansion and forest transitions from grasslands. Climate-driven changes in rainfall erosivity (R factor) also influence RV distribution, as it is a key driver of soil loss.

Finally, the Shannon Index (**SI**), which measures ecosystem diversity, is higher in areas with diverse ecosystem types, such as forests, grasslands, glaciers, and croplands. These areas are predominantly located in the northeastern part of the study area and the central part of the Adige River basin. By contrast, SI values are lower in homogeneous landscapes, such as large, forested regions or monoculture-dominated agricultural zones. The spatial distribution of SI remained relatively stable across the years, except for some sub-basins in the northwest under the SSP5-RCP8.5 scenario; here the expansion of forest is leading to a less diversification of land cover, thus decreasing the SI values. Although forests provide essential habitat for many species, extensive and continuous tree cover can lead to lower landscape heterogeneity, reducing overall ecosystem diversity. While this process can be beneficial for specific ESs such as carbon storage, it may simultaneously reduce the provision of other ESs and lead to the emergence of trade-offs.

### ESs bundles across different scenarios

**Fig. 4:**
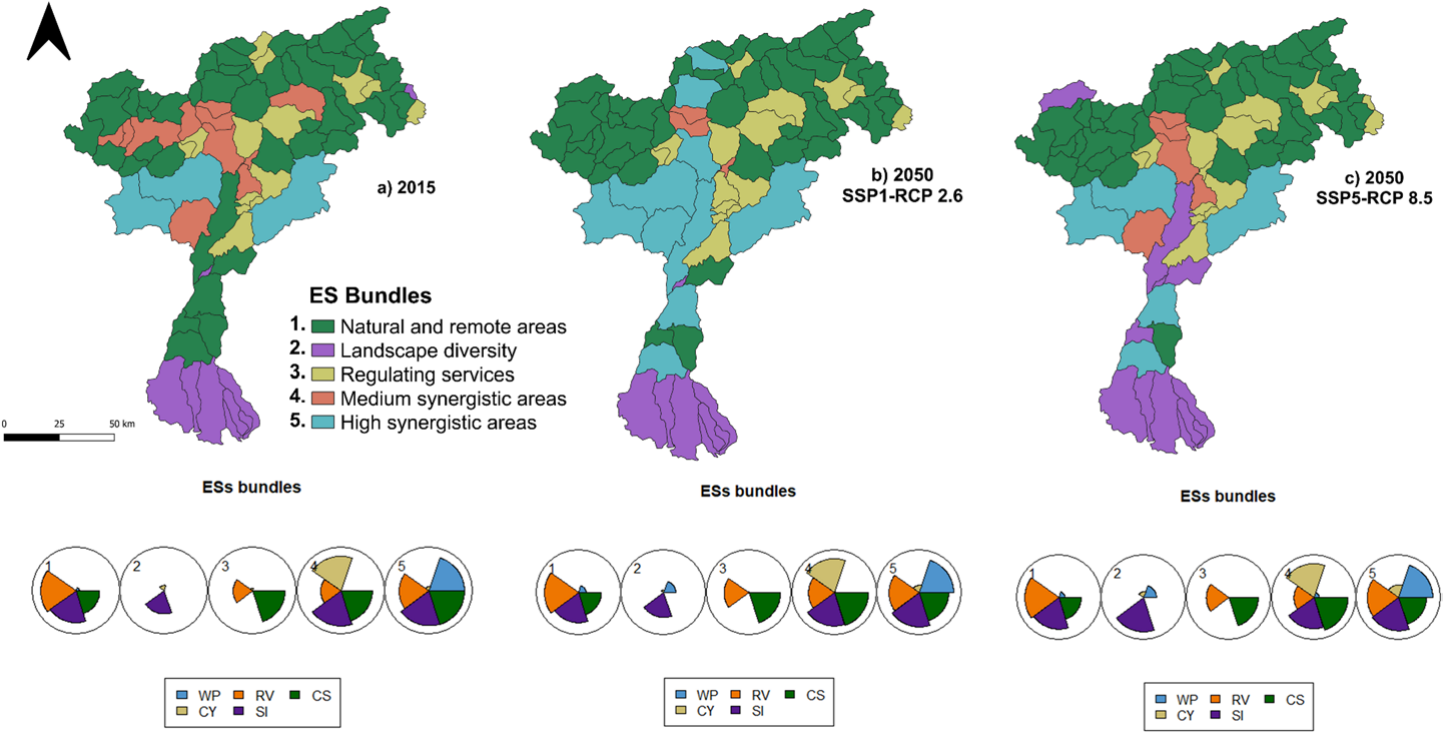
Ecosystem services bundles for the three scenarios under analysis. In the maps the spatial-temporal patterns of ES bundles are described, each color representing a different bundle that characterizes the sub-basins. In the graphs below composition and magnitude of ESs in ES bundles are reported, each color refers to the different ESs considered in the analysis, the segment represents the magnitude of ESs in each bundle, longer segments represent high ESs provisioning

**Fig. 5:**
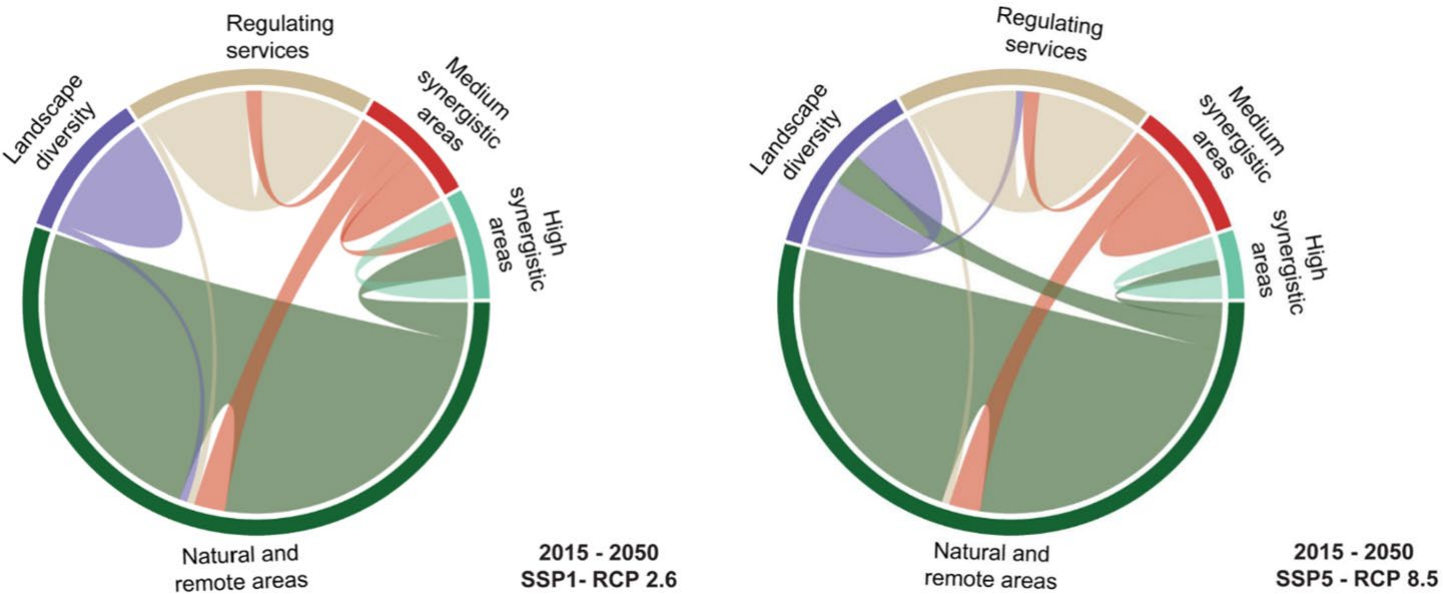
Bundles transition from baseline (2015) to future scenario (2050 SSP1-RCP 2.6) and future scenario (2050 SSP5-RCP 8.5)

SOM and manual tests identified five different ESs bundles (Fig. 4) at the sub-basin level in the whole Adige River basin. Figure 5; Table 3 describe the different ESs bundles developed with the SOM, providing a representation the specific ESs patterns characterizing each bundle and their spatial distribution in the three considered scenarios (i.e. 2015, 2050 SSP1-RCP26, 2050 SSP5-RCP85). The first bundle (**B1 – Natural and remote areas**) is mainly located in the northern part of the case study area (Fig. 4), characterized by mountainous landscapes with bare rocks, glaciers, perennial snow, and extensive forests (Fig. 1). It is defined by high values of soil retained by vegetation (RV), carbon storage (CS), and Shannon index (SI) indicators, indicating diverse natural ecosystems, such as forests, grasslands, pasturelands, and other types of natural vegetation. The presence of glaciers and snowpack plays a vital role in regulating water flow in the river basin, while the forested regions support various flora and fauna, enhancing biodiversity and contributing to essential ecosystem services.

**Table 3:**
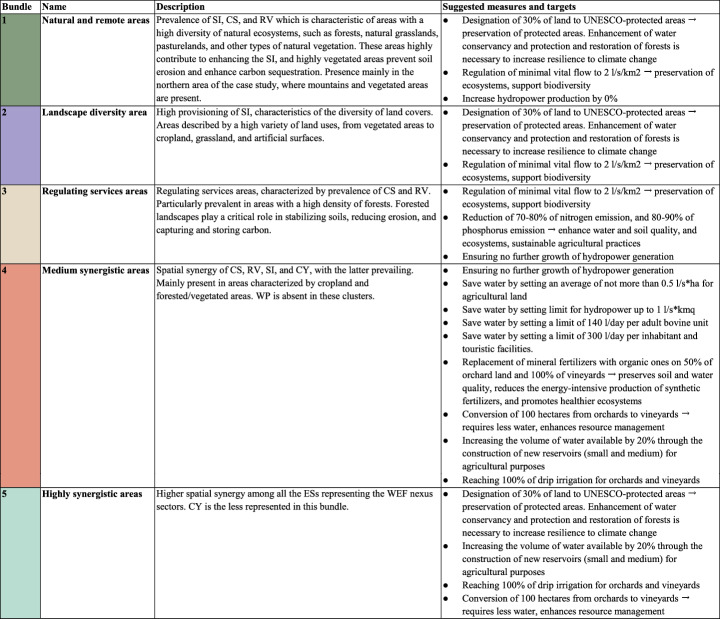
ESs bundles description

The number of the bundle, related name, description, measures and targetslinked to each bundle. Note that the targets provided are intended purely as normative benchmarks. As they are not based on empirical data or statistical analysis, no confidence intervals are provided

Bundle 2 (**B2 – Landscape diversity**), detected in the southern areas, is characterized mainly by a high SI, reflecting a mix of land covers including cropland, grassland, and artificial surfaces. As illustrated in Fig. 1, the mosaic of habitats contributes to landscape heterogeneity, supporting. a wider range of species and ecological interactions.

The third bundle (**B3 – Regulating services**) is less widespread across the sub-basins, but aligns with areas dominated by forests. It is characterized by high levels of regulating services such as carbon storage (CS) and soil retained by vegetation (RV).Forested regions play a critical role in stabilizing soils, reducing erosion, and capturing and storing carbon. While these vegetated areas support B3´s core services (CS and RV), the presence of non-contributing land covers like bare rock and glaciers limits the bundle’s spatial extent, confining it to smaller portions of the study area.

Bundle 4 (**B4- Medium synergistic areas**) can be found in the north-eastern part of the case study, where forest, mountains, vineyards area are present; it is characterized by a spatial synergy of CS, RV, SI, and CY. This bundle is in the areas where the mentioned ESs are mainly detected, reflecting the absence of WP (Fig. 3); these areas are characterized by forests which contribute to both CS and RV, but also in areas where agriculture is implemented, highlighting the importance of integrating forest conservation with agricultural practices to enhance overall ecosystem resilience and productivity. Moreover, having different types of land covers, SI is increasing, reflecting greater biodiversity and ecological complexity within the area.

The last bundle **(B5- High synergistic areas)** is localized in the central part of the study area, where multiple ESs show strong spatial synergy across the WEF nexus sectors, except for crop yield (CY), which is less pronounced. B5 is mainly seen in those sub-basins with high ESs provisioning (Fig. 3) and where different land covers (Fig. 1), including bare rock, forest, croplands, grassland, and, in particular glaciers and snow. Glaciers, and snow, are particularly important for regulating water provisioning (WP) and supporting hydrological processes, from which forests and natural areas take advantage and other ESs such as carbon storage (CS) and soil retention (RV) can benefit. These water sources also sustain downstream agricultural and energy sectors, reinforcing the overall synergy within the WEF nexus. This spatial configuration underscores the importance of managing these multifunctional landscapes to optimize ESs provisioning while balancing sectoral synergies and trade-offs.

Figure 5 represents the transition of each ESs bundle from baseline to different scenarios, showing how each bundle is converted into another and in which size these are present. From 2015 to the future scenario of 2050 under SSP1-RCP 2.6, both the maps and transition graphs (Figs. 4 and 5) indicate that Bundle 1 is expanding, while Bundle 2, Bundle 3, and Bundle 4 are shifting toward Bundle 1. In the northern part of the case study, Bundle 1 is particularly expanding in the SSP1-RCP 2.6 scenario, where there is a noticeable reduction in cropland areas together with an increase in bare rock, forest, and mountainous regions, which are mainly considered natural and remote areas. Additionally, some regions within Bundle 1 are transitioning toward Bundle 5, representing high synergistic areas where multiple ESs related to the WEF nexus coexist both in time and space. In the 2050 SSP5-RCP 8.5 scenario (Figs. 4 and 5), Bundle 1 moves towards Bundle 2, especially in the central region of the case study (Trento), where various types of land cover contribute to a higher Shannon index (SI). Bundle 1 also slightly shifts toward Bundle 5, indicating the increase of more ESs associated with WEF nexus sectors, where different land uses are located which contribute to providing these ESs, such as grassland, cropland, and forest Bundle 2 is transitioning towards Bundle 1 in SSP1-RCP 2.6, losing only one sub-basin (Fig. 3), while the expansion of mountainous areas and glacier melt leads to a decline in land cover diversity. In SSP5-RCP 8.5, Bundle 2 expands in the central part of the case study, increasing land cover types in this scenario and resulting in a higher SI. Bundle 3 exhibits slight expansion in both scenarios, particularly in the northern section of the case study area, where Bundle 2 and Bundle 4 were previously located. This change is most evident in areas where forest and vegetation have expanded, as Bundle 3 is closely linked to carbon sequestration (CS) and retained vegetation (RV), which are ESs strongly associated with this land cover type (Fig. 3). Bundle 4 ranks as the lowest bundle across all scenarios and is gradually being replaced by Bundle 2 and Bundle 5. Conversely, Bundle 5, representing areas where multiple ESs co-occur in time and space, shows notable expansion in the 2050 SSP1-RCP 2.6 scenario, particularly in mountainous regions, driven by the increase in forest land cover. Under the ‘*best-case scenario*’ (SSP1-RCP 2.6), it is possible to observe an expansion of high synergistic areas (Bundle 5), mainly driven by the transformation of natural and remote bundle (Bundle 1), as well as medium synergistic one (Bundle 4); from the point of view of WEF nexus this represents a situation where ESs are in synergies among each other, and they can be managed together to improve their provisioning. While in the ‘*worst case scenario*’ (SSP5-RCP 8.5), despite compared to the baseline (2015) Bundle 5 is increasing, this occurs only in central-southern areas. In both scenarios, Bundle 4 is decreasing, giving space to both Bundle 1 and 5, for ecosystems, CS, and natural habitats this is a good perspective, they’re increasing in provisioning of regulating services, but there could be disadvantages for provisioning ones (i.e. WP and CY).

### Identification of priority measures and targets for WEF nexus management

For each bundle, various management measures, and associated targets, have been proposed based on their potential to enhance ESs provision and support the different ESs within each bundle (Table 3) under different land use and climate change scenarios. These targets, derived from sectoral policies, serve as normative benchmarks for sustainable management, rather than empirically validated thresholds, and thus do not include confidence intervals. The proportions of forest, grassland, and cropland strongly influence the provision of CS and RV (Figs. 1 and 3), highlighting the need to preserve and expand vegetated areas. Designating 30% of land as a protected can enhance soil and water conservation, and forest restoration is necessary to increase resilience to climate change (European Commission 2022). This strategy is benefits Bundles 1 (forested and mountainous areas), 2 (diverse landscapes) and 5 (high ESs provision). The regulation of minimum vital flow setting the limit to 2 l/s/km2 plays a critical role in the preservation of ecosystems and the support of biodiversity. This flow standard ensures that even during periods of reduced water availability, rivers, and streams maintain a minimum water level necessary to sustain. Moreover, a regulated minimal flow contributes to the health of riparian zones, which are crucial for nutrient cycling, soil stabilization, and providing habitats for a variety of plant and animal species (Esquivel et al. 2020). This strategy can be applied both to Bundles 1 (preservation of all those natural areas), 2 (to support biodiversity) and 3 (to preserve those habitats fundamental in the provision of CS and RV). The disruption of natural hydrological cycles, largely due tohydropower development over the past century (Pérez Ciria et al. 2019), warrants halting further hydropower development and setting flow limits (in liters per second) to reduce erosion and water scarcity (Xia et al. 2023). This applies to Bundles 1(to preserve ecosystems and soil erosion), 3 (to mitigate soil erosion), and 4 (to preserve habitats, and ESs). From Fig. 3, it is evident that Bundle 4 (medium synergistic area) lacks water provisioning (WP) in its bundle description. Therefore, implementing measures to enhance WP could help conserve water and allow this ES to co-occur with others in both time and space. In Bundles 4 and 5, converting 100 ha of orchards to vineyards, crops that require less water, can improve landscape diversity and resource management. Replacing 50% of fertilizers with organic alternatives for orchards and 100% for vineyards can reduce emissions and improve soil health, as these are the most significant crops in the Adige River basin. The use of organic fertilizers can help decrease the energy-intensive production associated with synthetic fertilizers and promote a healthier ecosystem. Additionally, implementing 100% drip irrigation can increase water efficiency and reduce environmental impact.; This method promotes sustainable agricultural practices by conserving water, enhancing crop yield, and minimizing the environmental impact of irrigation on surrounding ecosystems. To improve water availability, constructing reservoirs to increase storage by 20% will support sustainable cropping and agricultural reliability. In Bundle 3, rich in regulating ESs like CS and RV, two measures are key: maintaining the 2 l/s/km² flow standard and reducing nitrogen emissions by 70–80% and phosphorus by 80–90% to enhance soil and water quality. These emission reduction strategies are also recommended for Bundle 2.

## Discussion

The spatial and temporal dynamics of ESs in the Adige River Basin reveal key trade-offs, particularly between provisioning and regulating services, driven by both climate and land use change. Crop Yield (CY) emerged as the most dynamic ES, showing projected increases in the northeastern and central sub-basins under future scenarios, primarily due to the expansion of high-value crops such as vineyards and orchards. This intensification reflects economic demands for stable food production, but it comes at the expense of ecosystem diversity (SI) and compromises long-term ecosystem resilience. Simultaneously, agricultural abandonment in alpine sub-basins leads to natural reforestation, enhancing Carbon Sequestration (CS) and Soil Retention (RV) but reducing open habitats and overall ecosystems diversity (SI). These dynamics mirror broader trends observed in the Trentino-Alto Adige region, as documented by Gobbi (2021) and Bonari et al. (2025). Studies by Cislaghi et al. (2019) and Mascetti et al. (2023) further emphasize that these land-use changes result in biodiversity loss and disrupted ecosystem functioning, particularly due to the disappearance of habitats critical for species dependent on open environments. This pattern is consistent with findings from Lasanta et al. (2017) in other mountain regions, where similar transitions have led to simplified landscapes and a substantial decline in the ecosystems’ ability to deliver multiple services and thus to a loss of multifunctionality of alpine landscapes. Additional trade-offs emerge from projected changes in hydrological services, particularly Water Provisioning (WP). Future scenarios indicate increasing climatic pressures and a decline in vegetated surfaces (e.g., expansion of bare rock), especially at higher altitudes, leading to reduced water yield (Rafiei-Sardooi et al. 2022). Diminished snowpack and altered precipitation regimes impact the natural regulation of river flows, intensifying competition among hydropower production, irrigation, ecosystem health, and drinking water supply (Siderius et al. 2022; Yin et al. 2023).

The ES bundles derived from the SOM analysis provide critical insights for guiding spatially differentiated policy responses to mitigate trade-offs and enhance synergies across the Adige River Basin. Their distinct compositions reflect underlying pressures from land use and climate change, emphasizing the need for flexible for site-specific strategies. (Saidi and Spray 2018).

For example, Bundle B2, marked by high landscape diversity and multifunctionality, is found in mosaic land use areas threatened by both agricultural intensification and forest sucession. These zones are essential for maintaining alpine habitat functions and should be prioritized for ecosystem restoration and protection, in line with the EU Restoration Regulation goals of restoring 20% of degraded ecosystems by 2030 and all by 2050, while fostering alignment with other EU climate strategies (e.g., the European Green Deal).

By contrast, Bundle B3, dominated by agricultural production, shows clear trade-offs with regulating services and declining landscape diversity. In such areas, sustainable transitions toward more sustainable agricultural practices considering agro-forestry, mixed cropping and the reduction of synthetic inputs in favour of organic fertilization (Muller et al. 2020 ), can help restore balance and improve soil and water quality. Promoting landscape diversity in agricultural land through organic farming and landscape mosaics, has been shown to strengthen ecosystem resilience and biodiversity (Brockerhoff et al. 2017). The designation of buffer areas, alongside targeted irrigation changes and emissions reductions, provides a pathway to harmonize human activities with natural systems (Gaglio et al. 2020), supporting both local well-being and global goals like the UN Sustainable Development Goals (Vörösmarty et al. 2021; Wood et al. 2018). Trade-offs between water uses are expected to intensify due to climate change. This is especially critical in medium-synergy areas (i.e. B4), where Water Provisioning (WP) is already low (Chiogna et al. 2016; Rafiei-Sardooi et al. 2022). Management options include the construction of reservoirs and the revision of environmental flow standards (Davis et al. 2015; Aznarez et al. 2021; Guo et al. 2022). Reduced reliance on water-intensive energy sources and integrated allocation is also crucial to preserve downstream ES flows and maintain riverine ecosystem health (Siderius et al. 2022; Yin et al. 2023).

Finally, high synergistic bundles like B5, where provisioning and regulating services co-occur, illustrate opportunities for the implementation multifunctional strategies that align with the WEF (Water-Energy-Food) nexus (Carmona-Moreno et al. 2021). These areas are suitable for practices such as agroforestry, rotational grazing, or mosaic landscape management, which can sustain productivity while preserving ecological integrity. In sub-basins where reforestation threatens biodiversity, conserving semi-natural grasslands and revitalizing traditional pastures can counteract landscape homogenization and sustain cultural services.

## Conclusion

Our study has contributed to understanding the spatial distribution and interactions of ESs within the WEF nexus in the Adige River basin. By analyzing five key ESs, water provisioning, crop yield, sediment retention, carbon storage, and landscape diversity, we show how climate and land use changes may reshape service provision across the basin. Our findings highlight the role of land cover and climate features in shaping ES dynamics and emphasize the need for integrated, landscape-specific management strategies. A key contribution is the use of SOM to identify and spatialize ESs bundles, enabling more targeted management interventions tailored to the diverse patterns observed across the bundles. For instance, some areas may require tailored policies to preserve and enhance ESs provisioning, particularly in scenarios where vulnerabilities and conflicts, such as those affecting crop yields, are evident. In the “best case ”scenarios, regions with synergistic ESs expand, supporting the WEF nexus, while “worst case ”scenarios reveal potential trade-offs requiring adaptive responses.

Our study highlights the limitations of simplified land cover data and the need for finer-scale research to capture more complex ESs processes, particularly under climate change. The spatial clustering of ESs into distinct bundles points to varied management priorities across the basin. However, the proposed measures, derived from existing sectoral policies, serve as normative sustainability benchmarks rather than empirically validated thresholds, and thus lack associated confidence intervals or measures of uncertainty. To make these strategies operational, future research should focus on testing and refining them under local conditions to assess their effectiveness and relevance to ecosystem dynamics.

As climate-driven shifts in temperature and precipitation reshape resource availability, integrated policy frameworks that consider the full spectrum of WEF nexus will become increasingly important. In conclusion, this research emphasizes the importance of multi-scale, ecosystem-based management to sustain critical services within the WEF nexus. The findings provide a foundation for future planning strategies that not only address individual ESs but also promote synergies that enhance the overall resilience of landscapes in the face of environmental change.

## Supplementary Information

Below is the link to the electronic supplementary material.

Supplementary Material 1
