# Supplementary material for: Managing the water-energy-food nexus in the adige river basin: impacts of climate and land use change on ecosystem services bundles
Source: Clim Change. 2025 Sep 11;178(9):168. doi: 10.1007/s10584-025-04013-3 (PMC12426146; doi:10.1007/s10584-025-04013-3)
Supplement: Supplementary file 1 — Supplementary Material 1 [file 10584_2025_4013_MOESM1_ESM.docx]

**Annex I - Self Organizing Maps**

ESs bundles are recurring sets of services that co-occur in space and time due to shared ecological, climatic, or socioeconomic drivers (Raudsepp-Hearne et al., 2010; Vannier et al., 2019). In this study, ESs bundles were identified using Self-Organizing Maps (SOM) by means of the “Kohonen” package (Willighagen et al., 2007) in R software, which provides tools for training and visualizing self-organizing maps. This allowed for the identification and visualization of ES bundles under the baseline and future scenarios, providing evidence to inform spatial management and planning strategies. To determine the optimal number of bundles, we conducted a series of manual tests using SOM, alternatively varying the number of clusters. The selection process aimed to maximize the interpretability and ecological distinctiveness of the resulting bundles while minimizing redundancy and overlap.

After a thorough evaluation of spatial coherence and ecological significance, the configuration with **five bundles** was selected as the most effective. This choice offered the best balance between differentiation and clarity, allowing for meaningful interpretation of the ecosystem service patterns without introducing unnecessary complexity.

In this Annex, we present three different clustering configurations, comprising four, five, and six clusters respectively, to illustrate the rationale behind selecting the five-bundle solution.

**Four Clusters (Figure IA)**: This configuration failed to capture the full range of ecosystem services under analysis. Some important services were either merged into broader, less informative categories or entirely overlooked, leading to a loss of potentially valuable ecological insights.


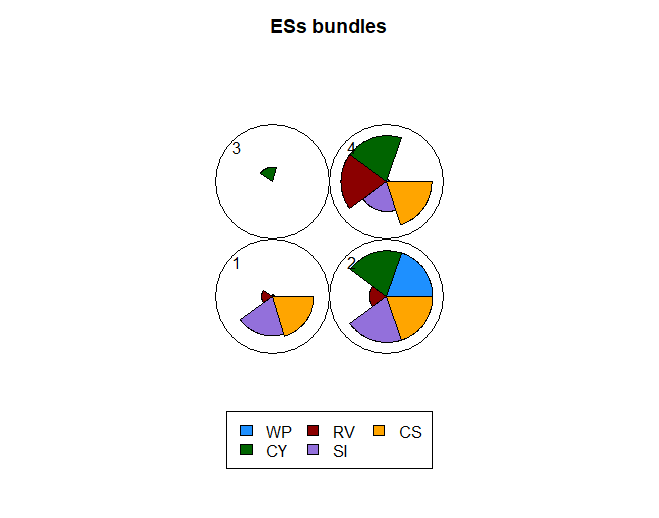


**Figure IA-Four Cluster configuration**

**Five Clusters (Figure IB)**: This setup provided a well-balanced representation of the ecosystem services. Each bundle was ecologically coherent and distinct, with minimal overlap. The services were grouped in a way that preserved their individual significance while highlighting meaningful spatial patterns.


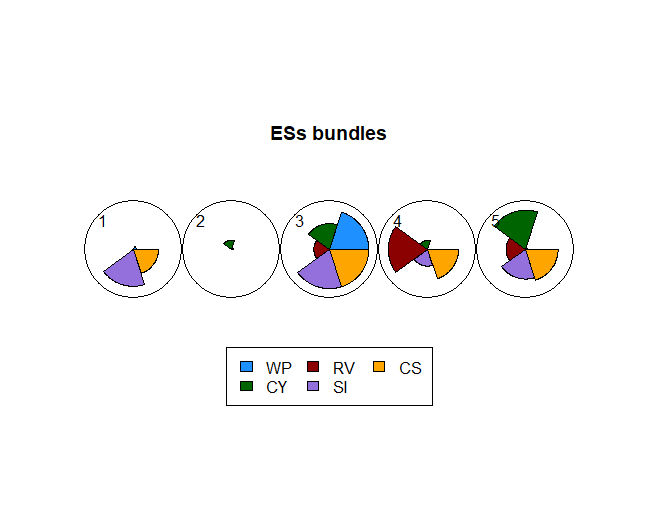


**Figure IB-Five Cluster configuration**

**Six Clusters (Figure IC)**: While this configuration offered slightly more granularity, it introduced redundancy. In particular, services such as soil retention by vegetation, the Shannon diversity index, and carbon storage appeared in multiple clusters with overlapping characteristics, reducing the clarity and interpretability of the results.


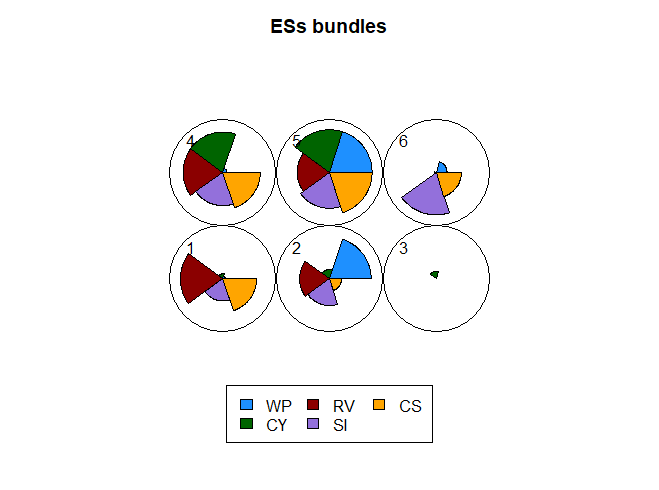


**Figure IC-Six Cluster configuration**

Overall, the five-cluster solution emerged as the most robust and interpretable, effectively capturing the complexity of the ecosystem services while maintaining analytical clarity.
